# Clinical outcome is associated with the Urinary Tract Dilatation Classification System grade

**DOI:** 10.3325/cmj.2020.61.246

**Published:** 2020-06

**Authors:** Petra Bratina, Tanja Kersnik Levart

**Affiliations:** 1 Obstetrics and Gynecology Hospital Postojna, Postojna, Slovenia; 2 Department of Nephrology, Division of Pediatrics, University Medical Centre, Ljubljana, Slovenia

## Abstract

**Aim:**

To assess the association between the Urinary Tract Dilatation (UTD) Antenatal (A) and Postnatal (P) Classification System grade and the outcome in term newborns.

**Methods:**

This retrospective study enrolled 166 term newborns (71% boys, 206 ureterorenal units) evaluated for unilateral or bilateral UTD in the Neonatology Department of Ljubljana University Medical Center from 2012 to 2018. Data on family history, sex, gestational age, birth weight, head circumference, Apgar score, possible oligohydramnios, indication for and age at first postnatal ultrasound, time of follow-up, and clinical outcome were collected. Radiology records were reviewed to grade UTD according to the Multidisciplinary Consensus on the Classification of Prenatal and Postnatal UTD.

**Results:**

The majority of ureterorenal units with UTD A 2-3 had UTD P 2 or 3. Spontaneous resolution, specific uropathy, the need for surgery, and the risk of urinary tract infection were all significantly associated with UTD P grade. No patient experienced renal dysfunction at the end of follow-up (12-48 months, median 24 months), and therefore this parameter was not associated with the UTD P grade.

**Conclusions:**

The UTD grade was associated with the probability of spontaneous resolution, time to its occurrence, specific uropathies, urinary tract infection, and risk for surgery. However, no association with renal dysfunction was established.

Urinary tract dilatation (UTD) is a relatively common finding in obstetrical ultrasound examinations (1%-5% of cases) (1-3). In the majority of patients, dilatation is transient or physiologic but can also occur due to obstructive or reflux conditions and lead to urinary tract infections, renal scarring, or renal dysfunction in postnatal life (4). It is, therefore, crucial to timely select those children who would benefit from early surgical intervention and, at the same time, not to overevaluate those with a low risk of renal complications.

Until 2014, several UTD grading systems were used (5-10), leading to the lack of uniformity in defining and grading UTD and questionable associations between prenatal and postnatal US findings and the ultimate urological diagnosis, not to mention the outcome. In 2014, Nguyen et al introduced a new, uniform classification system that can be used across the prenatal (A) and postnatal (P) time period to describe UTD and facilitate communication between different specialists (4). This postnatal grading system is well correlated with the risk of postnatal uropathies (4), and UTD P grading is associated with other clinical outcomes, such as spontaneous resolution (11), urinary tract infections (UTI), and the need for surgical intervention (12). There are, however, no firm data on UTD A grading and its association with these clinical outcomes.

The aim of our study was to assess the association of different UTD P grades, as defined by the UTD P grading system proposed by Nguyen et al (4), with specific clinically relevant outcomes. In addition, we assessed the association of different UTD A grades to UTD P grades. We hypothesized that the majority of ureterorenal units with UTD A 2-3 would be classified into the UTD P 2 or 3 group. If this is the case, then the association of UTD A with clinically relevant outcomes would be the same as the association of UTD P with these outcomes. We further hypothesized that there was an association between UTD P grade and the probability of spontaneous resolution, time to its occurrence, specific uropathies, urinary tract infection, the risk for surgery, and renal dysfunction.

## Patients and methods

This retrospective study enrolled 166 term newborns (206 ureterorenal units) evaluated for unilateral or bilateral UTD at the Division of Pediatrics, Neonatology Department, University Medical Centre, Ljubljana, Slovenia, from 2012 to 2018. The inclusion criteria were term birth and prenatally or postnatally detected unilateral or bilateral UTD.

The indications for postnatal ultrasound (US) of the urinary tract were prenatal or previous postnatal urinary tract US examinations showing unilateral or bilateral UTD, suspected infections (early or late-onset sepsis, urinary tract infections), other syndromes (screening due to other congenital anomalies/syndromes or known familiar urinary tract anomalies), and positive family history. The study was approved by the National Medical Ethics Committee (71/11/11).

Initially we analyzed prenatal US data and determined the UTD A grade according to the Multidisciplinary Consensus on the Classification of Prenatal and Postnatal UTD (4) as follows:

- UTD A 1 – low risk of postnatal uropathies,

- UTD A 2-3 – increased risk of postnatal uropathies.

Later we analyzed the US examinations performed from 2012 to 2018 by a trained pediatric radiologist blinded to the study aim on an Aplio XL US machine (Toshiba Corp., Tokyo, Japan) with linear (4.5-7.5 MHz), micro convex (5.0-7 MHz), and convex (2.5-5 MHz) probes. Urinary tract US was considered normal if the kidney length (between 5th and 95th percentile) and shape were normal, if there was no dilatation of the ureter and pelvicalyceal system, and if the renal parenchyma, and the kidney and bladder echogenicity were normal. On the basis of US examinations analysis, UTD was graded according to the Multidisciplinary Consensus on the Classification of Prenatal and Postnatal UTD as (4):

- UTD P1 – low risk of postnatal uropathies;

- UTD P2 – intermediate risk of postnatal uropathies;

- UTD P3 – high risk of postnatal uropathies.

We collected the data on the family history of congenital anomalies of the kidney and urinary tract (CAKUT), sex, gestational age, birth weight, head circumference, Apgar score, possible oligohydramnios, indication for and age at first postnatal US, follow-up length, and clinical outcome (spontaneous resolution, time to its occurrence, urologic diagnosis, need for surgery and age at surgery, occurrence of urinary tract infection, and renal dysfunction at the end of follow-up).

Spontaneous resolution was defined as a clinically normal follow-up US. The etiology of UTD, determined on the basis of other diagnostic investigations (voiding cystographies, scintigraphies), was vesicoureteral reflux (VUR), ureteropelvic junction obstruction (UPJO), ureterovesical junction obstruction/megaureter, posterior urethral valve, combinations, and other (ureterocele). Surgery was performed when the following indications were met: worsening of UTD and/or an obstructed renogram with differential renal function <35%-40% or its subsequent decline; recurrent febrile UTI in VUR despite antibiotic prophylaxis and/or decline in differential renal function. An UTI was considered to be present when a child had at least a positive urinary culture (more than 10^5^ colony forming units/mL) and pyuria in urine collected by pediatric urine collector (13). Renal dysfunction was defined by reduced glomerular filtration rate, which was estimated from the serum creatinine concentration using the Schwarz formula: eGFR = height (cm) × 0.45/s-creatinine (mg/dL) (mL/min/1.73m^2^) (14).

### Statistical analysis

Categorical variables are expressed as frequencies and percentages, and numerical variables are expressed as medians and interquartile ranges. The significance of association between categorical parameters was assessed with the χ^2^-test or Fisher exact test. The Kruskal-Wallis test was used for scale parameters and long-rank test (survival analysis using Kaplan-Meier method) for censored data. The McNemar test was used to determine the sensitivity and specificity of the prenatal and postnatal UTD classification. The level of significance was set at *P* < 0.05. The statistical analyses were performed with Microsoft Excel (Microsoft, Redmond, WA, USA) and SPSS software, version 24.0 (IBM, Armonk, NY, USA).

## Results

### Patients'/ureterorenal units' characteristics according to prenatal US and association of UTD A to UTD P grade

A hundred and twenty-six patients had unilateral, while 40 had bilateral UTD. A total of 19.3% patients had positive family history of CAKUT. Patients in different UTD groups did not differ significantly in gestational age, birth weight, head circumference, Apgar score, age at first postnatal US, and follow-up period. Oligohydramnios was found only in children with UTD A 2-3 and non-defined UTD A grade. The majority of ureterorenal units (88.3%) with UTD A 2-3 had UTD P 2 or 3 (sensitivity 88%, specificity 32%; McNemar test, *P* < 0.005) (Table 1).

**Table 1:** Characteristics of patients/ureterorenal units according to prenatal ultrasound*

	All	No UTD A	UTD A1	UTD A2-3	Non-defined UTD A grade	P
Ureterorenal units (N)/patients (n)	206/166	26/24	12/8	43/37	125/97	
Positive family history (n/%)	32/166 (19.3)	5/24 (20.8)	2/8 (25.0)	5/37 (13.5)	20/97 (20.6)	0.740^†^
Sex (n/%)						
male	118 (71.1)	17 (70.8)	2 (25.0)	25 (67.6)	74 (76.3)	0.019^‡^
female	48 (28.9)	7 (29.2)	6 (75.0)	12 (32.4)	23 (23.7)	
Gestational age (weeks), median (IQR)	39 (38-40)	40 (39-40)	40 (39-40)	39 (38-40)	39 (38-40)	0.165^§^
Birth weight (g), median (IQR)	3455 (3160-3810)	3575 (3160-3998)	3365 (3145-3738)	3410 (3140-3900)	3460 (3195-3800)	0.934^§^
Head circumference (cm), median (IQR)	35 (34-36)	36 (34-36)	35 (34-36)	35 (34-36)	35 (34-36)	0.693^§^
Apgar score (at 5 min), median (IQR)	9 (9-10)	9 (9-9)	10 (9-10)	9 (9-10)	9 (9-10)	0.154^§^
Oligohydramnios (n/%)	7/166 (4.2)	0/24 (0.0)	0/8 (0.0)	1/37 (2.7)	6/97 (6.2)	0.743^†^
Indications for postnatal US						
pathological US-A	139/166 (83.7)	3/24 (12.5)	8/8 (100.0)	37/37 (100.0)	94/97 (96.9)	
suspected UTI	12/166 (7.2)	10/24 (41.7)	0/8 (0.0)	0/37 (0.0)	2/97 (2.1)	
pathological US-P	5/166 (3.0)	4/24 (16.7)	0/8 (0.0)	0/37 (0.0)	1/97 (1.0)	
other syndromes	3/166 (1.8)	3/24 (12.5)	0/8 (0.0)	0/37 (0.0)	0/97 (0.0)	
positive FH	3/166 (1.8)	3/24 (12.5)	0 / 0.0	0/37 (0.0)	0/97 (0.0)	
unknown	4/166 (2.4)	4/24 (16.7)	0 / 0.0	0/37 (0.0)	0/97 (0.0)	
Age at first US (days), median (IQR)	5 (3-10)	5 (3-13)	5 (4-12)	4 (3-7)	6 (3-10)	0.526^§^
UTD P (N/%)						
1	40/206 (19.4)	5/26 (19.2)	3/12 (25.0)	5/43 (11.6)	27/125 (21.6)	0.182^‡^
2	70/206 (34.0)	11/26 (42.3)	7/12 (58.3)	13/43 (30.2)	39/125 (31.2)	
3	96/206 (46.6)	10/26 (38.5)	2/12 (16.7)	25/43 (58.1)	59/125 (47.2)	
Follow-up (months), median (IQR)	24 (12-48)	24 (17-47)	16 (11-35)	24 (12-44)	25 (12-48)	0.631^§^

*UTD – urinary tract dilatation; A – antenatal; P – postnatal; N – number of ureterorenal units; n – number of patients; IQR – interquartile range; US – ultrasound; FH – family history; UTI – urinary tract infection.

^†^Fisher exact.

‡χ^2^ test.

§Kruskal-Wallis.

### Association between different UTD P grades and specific outcomes

The occurrence of spontaneous resolution was significantly associated with the UTD P grade (*P* < 0.001). Spontaneous resolution was most common in the UTD P1 group and least common in the UTD P3 group. In addition, the time to spontaneous resolution was significantly shorter in the lower UTD P grade groups (*P* = 0.011).

The occurrence of specific uropathy was significantly associated with the UTD P grade (*P* < 0.001). The most common uropathy in the UTD P3 group was UPJO, while the most common uropathy in the UTD P2 and P1 group was VUR.

The need for a urologic procedure was significantly associated with the UTD P grade (*P* < 0.001). No urologic procedure was needed in the UTD P1 group, while 62.5% participants in the UTD P3 group (the most commonly needed procedure was pieloplasty) and 14.3% in the UTD P2 group needed a urologic procedure. The age at surgery did not differ among the groups. However, this was not the case among different groups of uropathies. In VUR, the need for surgery was not associated with UTD P grade, while in other uropathies it was, and surgery was most commonly performed in the UTD P3 group.

The risk of UTI was significantly associated with the UTD P grade (*P* < 0.003) and was highest in patients with UTD P3 and lowest in those with UTD P1.

No patient experienced renal dysfunction at the end of follow-up (12-48 months, median 24 months), while in 3 patients with UTD P3 no data on renal function were available (Table 2).

**Table 2:** Association between different grades of urinary tract dilatation and specific outcomes*

	No. (%) of ureterorenal units				
	All N = 206	UTD P1 N = 40	UTD P2 N = 70	UTD P3 N = 96	P
Spontaneous resolution	70/206 (34.0)	28/40 (70.0)	32/70 (45.7)	10/96 (10.4)	<0.001^†^
Time to resolution (months), median (IQR)	12 (6-17)	9 (3-12)	13 (6-21)	24 (7-36)	0.011^‡^
Specific uropathies					<0.001^†^
VUR	43/206 (20.9)	4/40 (10.0)	22/70 (31.4)	17/96 (17.7)	
Other					
UPJO	43/206 (20.9)	3/40 (7.5)	5/70 (7.1)	35/96 (36.5)	
UVJO	15/206 (7.3)	0/40 (0.0)	3/70 (4.3)	12/96 (12.5)	
PUV	2/206 (1.0)	0/40 (0.0)	0/70 (0.0)	2/96 (2.1)	
combinations	15/206 (7.3)	0/40 (0.0)	3/70 (4.3)	12/96 (12.5)	
ureterocele	3/206 (1.5)	0/40 (0.0)	0/70 (0.0)	3/96 (3.1)	
no specific uropathy	85/206 (41.3)	33/40 (82.5)	37/70 (52.9)	15/96 (15.6)	
Renal surgery	70/206 (34.0)	0/40 (0.0)	10/70 (14.3%)	60/96 (62.5)	<0.001^†^
the Cohen procedure	8/206 (3.9)	0/40 (0.0)	1/70 (1.4)	7/96 (7.3)	
subureteric injection of Vantris	4/206 (1.9)	0/40 (0.0)	2/70 (2.9)	2/96 (2.1)	
pyeloplasty	40/206 (19.4)	0/40 (0.0)	4/70 (5.7)	36/96 (37.5)	
PUV resection	2/206 (1.0)	0/40 (0.0)	0/70 (0.0)	2/96 (2.1)	
nephrectomy	12/206 (15.8)	0/40 (0.0)	3/70/ (4.3)	9/96 (9.4)	
other	3/206 (1.5)	0/40 (0.0)	0/70 (0.0)	3/96 (3.1)	
unknown	1/206 (0.5)	0/40 (0.0)	0/70 (0.0)	1/96 (1.0)	
Age at surgery (months), median (IQR)	4 (2-8)	/	5 (2-18)	4 (2-7)	0.575^§^
	**No. (%) of patients**				
	**All** n = 166	**UTD P1** n = 32	**UTD P2** n = 53	**UTD P3** n = 81	
UTI	68/166 (41.0)	6/32 (18.8)	24/53 (45.3)	38/81 (46.9)	0.003^†^
0	98/166 (59.0)	26/32 (81.3)	29/53 (54.7)	43/81 (53.1)	
1	39/166 (23.5)	2/32 (6.3)	15/53 (28.3)	22/81 (27.2)	
2	7/166 (4.2)	2/32 (6.3)	2/53 (3.8)	3/81 (3.7)	
3 or more	22/166 (12.3)	2/32 (6.3)	7/53 (13.2)	13/81 (16.0)	

*UTD – urinary tract dilatation, P – postnatal, N – number of ureterorenal units, n – number of patients, IQR – interquartile range, VUR – vesicoureteral reflux, UPJO – ureteropelvic junction obstruction, UVJO – ureterovesical junction obstruction, PUV – posterior urethral valve, UTI – urinary tract infection.

†χ^2^ test.

‡Log-rank.

§Kruskal-Wallis.

## Discussion

Our results showed that the majority of ureterorenal units with UTD A 2-3 had UTD P 2 or 3. Spontaneous resolution, specific uropathy, the need for surgery, and the risk of urinary tract infection were all significantly associated with the UTD P grade.

The first study to assess the reliability of the UTD P system in predicting outcomes was that by Hodhod et al (15). According to their findings, UTD P grade can predict the resolution rate of hydronephrosis. Our study also showed that spontaneous resolution was most common and fastest in UTD P1. Similarly, Nelson et al (16) reported UTD resolution in 41% of participants in a median of 10.1 months (in our study in 34% participants in a median of 12 months) and a significant variation of UTD resolution by UTD grade. In concordance with their results, our study also reported a significant variation in the rate of spontaneous resolution – 70/45.7/10.4% in UTD P1/2/3, respectively. In contrast, Braga et al (11) detected much lower variation (90/81/71% in UTD P1/2/3, respectively), which could be explained by the exclusion of children with surgery from their study.

The association between high UTD grades and UTI risk is rarely described in the literature (15). We showed the risk of UTI to be highest in patients with UTD P3. Moreover, the children in this group had the highest incidence of recurrent UTI.

Our study, as well as that by Nelson et al (16), showed that the need for urologic procedure was significantly associated with the UTD P grade. The overall 34% surgery rate in all UTD groups in our study was, however, higher than 12% reported by Hodhod et al (15). Furthermore, our study showed that the association between the need for surgery and the UTD P grade was not consistent among different uropathy groups. In the case of VUR, the need for surgery was not associated with the UTD P grade, while in the case of other uropathies it was, with the surgery being most commonly performed in the UTD P3 group.

There are no reports on the association of UTD grade and renal dysfunction at the end of follow-up. In our study, no patients had renal dysfunction; however, patients were followed for 12-48 months (median 24 months), meaning that the results might have been affected by the follow-up length.

We are well aware that the study’s retrospective design and the single-center inclusion of patients, and therefore their limited number, create an implicit bias. However, we believe that owing to its comprehensive approach, this study adds value to the literature. Due to the retrospective data analysis, more than half of the ureterorenal units were classified as non-defined UTD A grade, since not all the necessary data to reliably classify the UTD A grade were available. In spite of this, we confirmed our first hypothesis and showed that one can very reliably, with a sensitivity of 88%, predict high UTD P grades from high UTD A grades. We, therefore, believe that to assess an association of UTD A grades with clinically relevant outcomes, one can, at least to some extent, use the association of UTD P grades to these outcomes. The Multidisciplinary Consensus postnatal urinary tract dilatation grading system is confirmed to be associated with the risk of postnatal uropathy. Our results and some other reports indicate that it is also associated with clinically relevant outcomes, such as the probability of spontaneous resolution, time to its occurrence, urinary tract infection, and risk for surgery. However, no association to renal dysfunction was found. The latter finding should be elucidated in more extensive studies with a longer follow-up.
